# Rural to Urban Migration Is an Unforeseen Impact of Development Intervention in Ethiopia

**DOI:** 10.1371/journal.pone.0048708

**Published:** 2012-11-14

**Authors:** Mhairi A. Gibson, Eshetu Gurmu

**Affiliations:** 1 Department of Archaeology and Anthropology, University of Bristol, Bristol, United Kingdom; 2 Center of Population Studies, College of Development Studies, Addis Ababa University, Addis Ababa, Ethiopia; Bristol University, United Kingdom

## Abstract

Rural development initiatives across the developing world are designed to improve community well-being and livelihoods. However they may also have unforeseen consequences, in some cases placing further demands on stretched public services. In this paper we use data from a longitudinal study of five Ethiopian villages to investigate the impact of a recent rural development initiative, installing village-level water taps, on rural to urban migration of young adults. Our previous research has identified that tap stands dramatically reduced child mortality, but were also associated with increased fertility. We demonstrate that the installation of taps is associated with increased rural-urban migration of young adults (15–30 years) over a 15 year period (15.5% migrate out, n = 1912 from 1280 rural households). Young adults with access to this rural development intervention had three times the relative risk of migrating to urban centres compared to those without the development. We also identify that family dynamics, specifically sibling competition for limited household resources (e.g. food, heritable land and marriage opportunities), are key to understanding the timing of out-migration. Birth of a younger sibling doubled the odds of out-migration and starting married life reduced it. Rural out-migration appears to be a response to increasing rural resource scarcity, principally competition for agricultural land. Strategies for livelihood diversification include education and off-farm casual wage-labour. However, jobs and services are limited in urban centres, few migrants send large cash remittances back to their families, and most return to their villages within one year without advanced qualifications. One benefit for returning migrants may be through enhanced social prestige and mate-acquisition on return to rural areas. These findings have wide implications for current understanding of the processes which initiate rural-to-urban migration and transitions to low fertility, as well as for the design and implementation of development intervention across the rural and urban developing world.

## Introduction

For the first time in human history, more than half the world's population lives in urban areas. Over 90 percent of urbanization is taking place in the developing world, and is particularly concentrated among young adults (aged 15–30 years) in Africa and Asia, the result of rapid population growth occurring during the mid twentieth century [1], [2]. Projections for Ethiopia, currently one of the least urbanized countries in the world, indicate that the proportion of people living in urban centres will double over the next forty years (from 17% in 2010 to 38% in 2050) [1].

In principle, cities offer a more favourable setting for tackling social and health problems than rural areas. Cities generate jobs and income, deliver education, health care and other services more efficiently than less densely settled areas, simply because of their advantages of scale and proximity [3]. However, in less developed countries such as Ethiopia, rapid urbanization is increasingly concentrating poverty, placing strain on infrastructure and already stretched public services in towns and cities [4]–[6]. A key challenge for the next century is managing the scale and pace of urbanization, particularly in countries which have fewer resources and slower rates of economic growth [7], [8].

Implicit in the debates about managing the pace of population growth and urban expansion is the recognition of increasing need for improved and integrated public services, which help to reduce rural poverty and unwanted fertility. Development intervention initiatives are designed to respond to these needs, often based around the introduction of new technologies to improve health and livelihoods in rural communities. However, many schemes operate in regions where family planning uptake is low, due to a lack of availability, or social opposition (e.g. in rural Ethiopia less than a quarter of women use “modern” contraception [9]). In the absence of contraception or desire for family limitation, rural development which improves health and well-being may be contributing to rather than relieving population pressure [10].

Our previous research revealed that reduction to workload has been the largest influence of a new water development initiative on women's lives in a rural Ethiopian community. The installation of village-level tap stands has reduced the time and effort that women spend carrying water on their backs; from 6 hours/day to less than 30 minutes in some instances. However, it has also introduced unforeseen demographic consequences [10], [11]. Access to water taps has been linked directly to an immediate increase in reproductive rates, supporting an evolutionary life history theory prediction that improvements in women's energy budgets would translate directly into higher fertility [12]. Women gaining access to taps are now four times more likely to give birth on a given month than those without access to taps. Combined with improvements in child survival, brought about by improved water supplies (reducing child death by 50% for every month of life), this has underpinned increases in family sizes in a population at carrying capacity [10]. In the context of population growth and resource scarcity, a number of negative consequences have been observed, including higher rates of childhood malnutrition [10] and maternal depletion in body condition [11]. We also found that discriminative investment in children's education was more pronounced in villages with tap-stands [13]. Here we extend this research to investigate patterns and motivations of rural to urban migration in the study villages, focussing on the influence of recent rural population changes brought about by the water tap installation on out-migration. It is predicted that rural development intervention has favoured increased rural to urban migration of young adults.

The complexity of the migration process, and range of push and pull factors which may influence the decision to migrate from rural to urban centres has been clearly demonstrated by demographers, economists and evolutionary anthropologists [14]–[17]. Migration is generally viewed as a strategy of risk avoidance and resource diversification, with costs and benefits which can be shared by the individual, household and the wider group. Risk avoidance could be from local resource shortages - e.g. sibling competition for heritable agricultural plots too small to sustain a family [18], [19]- or where political upheaval or climate change threatens local livelihoods [20], [21]. Resource diversification is anticipated through new income-generating opportunities and improved access to education available in urban centres [22], [23]. Rural out-migration incurs costs, both in money and time spent away from rural subsistence tasks, but economic remittances, the flow of money back to the household and improved social capital and networks are all perceived to reduce the costs of migration and increase the resilience of the rural household [16], [24], [25]. For example, a recent study found that rural Ethiopian households receiving migrant remittances were less likely than other households to sell productive assets, such as livestock, to cope with food shortages [26].

A key assumption is that the incentives to migrate are influenced by the composition and behaviours of other members of the household. For example, the arrival of new offspring may place an additional strain on scarce resources (such as food or land); or the departure of one member should create opportunities for others to move out (through chain migration). Family dynamics are assumed in both evolutionary and economic studies, but often understudied in these contexts. Here we extend the literature to explore how the exact timing of household members' movements may influence an individual's risk of migrating out.

This study has been designed to exploit a unique natural experiment that uses longitudinal survey data collected before and after the installation of water taps across a sample of otherwise comparable Ethiopian villages. We are able to test both the direct impact of development and family dynamics on the probability of out-migration over-time. Multilevel hazards regression modelling analyses explore migration in relation to a range of time-varying social and demographic phenomena for each individual, addressing the impact of 1) the arrival of the new development technology, 2) the arrival of a new birth in the household, 3) the arrival of a spouse in the household, 4) the departure of a child in the household, and 5) the departure of an adult from the household. In addition to measuring levels of migration, we explore stated reasons for movement, as well as the value of migration through remittances and marriage.

### Study site

**Figure 1:**
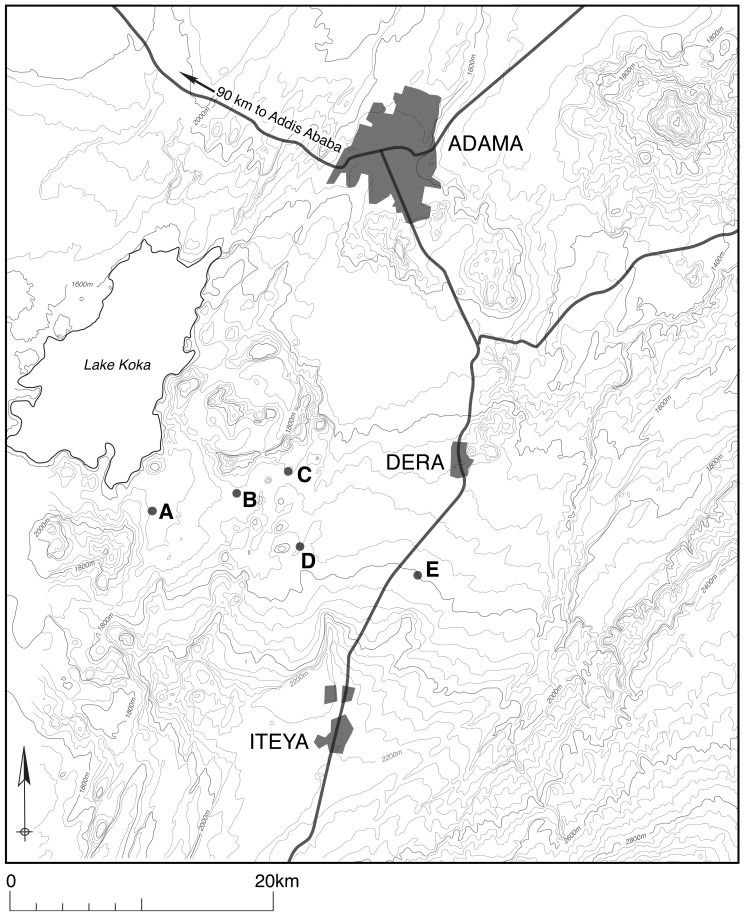
Map of study site.

This study is based on a rural Arsi Oromo agropastoralist community in Southern Ethiopia (see map in Figure 1), which suffers from acute, regular water shortages and chronic food insecurity, as well as recent population growth. A declining ratio of land to people, lower agricultural productivity, and limited infrastructure (no electricity, few schools and health services) are features prevailing in this rural community. Few off-farm employment or income generation opportunities exist: less than 10% of households in the study population reported any earnings from non-agricultural activities in the survey year (2009). Schooling opportunities in the villages are limited to primary level, and many adults remain uneducated (35% of males and 75% of females have never attended school).

**Figure 2:**
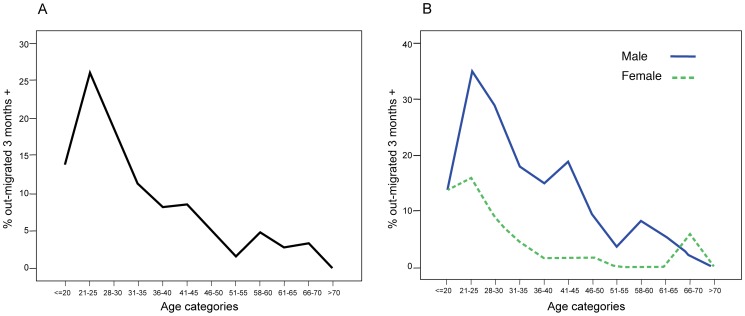
Percentage rise in adults ever-migrated out by age categories n = 3698 (all household members >15 years). Section A includes all adults, and Section B, by sex.

Use of family planning is limited (less than 20% women have used contraception); the average woman has 8 births during her lifetime, a quarter of these children dying within the first 5 years of life. In recent years increases in agricultural land scarcity, together with few local economic opportunities, have led to a rise in the number of adults migrating to urban centres (rising from <5% to 20% over the last 50 years: Figure 2). This movement has been greatly facilitated by broad infrastructural improvements in transport and communication links to nearby towns, e.g. improved all-season roads and mobile phone reception, and access to modern farming equipment, which has reduced the need for agricultural labour.

However, between 1996 and 2000 some villages in the region benefited from a new rural development initiative with the primary goal of improving access to a safe and reliable water supply. The distribution of villages with taps was determined by arbitrary administrative boundaries rather than local demand (for village characteristics see Table 1). The installation of village-level taps has reduced the time and energy women spent carrying water on their backs [27]. In addition it has had an immediate and positive effect on childhood survivorship, reducing risk of child death by 50% per month of life, and increased birth rates in the absence of family planning, resulting in a four-fold increased relative risk of child birth per month for women gaining access to the new labour-saving technology [10], [11]. Increases in family size have placed greater pressure on intrahousehold resources – introducing high rates of childhood malnutrition [10], and inequalities in access to education [13].

**Table 1:** Village characteristics.

	A	B	C	D	E	Total
Population size	1637	1297	1337	1648	1119	7038
Number of households	291	253	222	312	202	1280
Sample (aged 15–30)	438	328	378	426	342	1912
Age	21.96 (±4.36)	22.88 (±4.46)	22.43 (±4.60)	22.40 (±4.56)	22.52 (±4.38)	22.41 (±4.48)
% Migrants	15.3%	11.5%	19.8%	13.3%	25.7%	17.5%
Years of education	4.37 (±3.4)	3.46 (±3.2)	4.98 (±3.5)	3.89 (±3.1)	5.28 (±3.7)	4.39 (±3.42)
% Landless	84.2%	89%	87.6%	88.5%	86.3%	87%
Date of access to development	2000	none	none	1996	1996	

## Methods

Migration histories, demographic, socio-economic and anthropological data were collected in 2009–10 in five Arsi Oromo villages, which included those with and without access to the water development scheme. Selection criteria for these villages included comparability of size, altitude, ethnicity, and religion, and distance from local towns and roads, facilitating comparative analyses both between and within villages (for village characteristics see Table 1 and Figure 1). The comparability of villages provided an opportunity for a natural experimental framework, allowing us to quantify migration rates both before and after tap installation, and across villages (including those with and without access to development) to assess the impact of this development intervention on the movement patterns of young adults.

### Data Collection

A broad demographic census and survey was completed in all households (n = 1280) providing data on major socio-economic and demographic factors that may influence the decision to migrate out (e.g. household wealth, household size, labour and religion). All household heads were interviewed providing full retrospective migration histories for all household members, including non-resident individuals who had migrated out temporarily or permanently. Migration did not include females who moved between local villages for marriage, common in this patrilocal society. Data collection included a detailed events calendar that recorded the timing of all movements of adults and children (<15 years of age) into and out of the household over the 15 years preceding the interview (1994–2009); hence, the periods before and after the water tap installation (1996 & 2000). Each life history event was dated to the year and to the season (a 3 month time period) using a calendar marked with local significant events. In addition, full information on earnings, flows of remittances and education was collated for each individual in the household. To understand local perceptions and attitudes, anthropological data collection was undertaken through focus group discussions with groups of men and women living in the villages.

For statistical analyses, the sample was restricted to 1,912 young adults, which included all aged 15–30 years within these households (including 996 males and 916 females). Young adults 15–30 years of age were selected as they represent those most likely to migrate out of rural areas, 15.5% of whom have ever-migrated out (Figure 2).

### Ethics Statement

Research and Ethical clearance was obtained from the University of Bristol Research Ethics Committee and the Addis Ababa University Ethics Committee - the Academic Commission of the College of Development Studies (Ref: CDS/AAU/191/09). Permissions were granted from Local and Regional Authorities in Ethiopia. All participants were adult head of households. Procedures were explained to each participant and signed or fingerprinted consent was obtained from all participants. Ethics committees were aware that minors (under 18 years) would provide their own consent.

### Analyses

Discrete-time event history analysis was used to assess the effects of the rural development initiative on the probability of out-migration of young adults. Event history analysis is a powerful statistical tool for isolating the precise timing of demographic effects, e.g., the date of access to development on the monthly risk of childbirth and child death. Unlike other standard forms of regression analysis, it can deal with both censored and time-series data (variables which change over time) [28], [29]. For each young adult, the beginning of time corresponds to the date they reached age 15 within the events calendar (1994–2009). Once they had migrated out, individuals exited the dataset. Those who had not migrated out by the survey date in 2009 were considered “right censored”. The probability that an individual will migrate out in a given season (3 month period) can be calculated, conditional on no prior event occurrence.

Models of relative probabilities of out-migration over time included fixed and time-varying variables. Fixed variables included both household and individual level data, such as village, religion, household wealth [land holdings and cattle herd size], head of household's education status [attended school or not], resident household size, sibship size, birth order and sex. See supplementary information Table S1 for a full list of variables and sample description. Since the exact timing of the tap installation was known, access to development intervention was entered into the model as a time-varying covariate [March 1996 or January 2000]. The timing of movements of other household members over the observation period was also entered as time-varying variables, including seasonal movements both into and out of the household. This included the arrival of a new spouse [adult arrives], the departure of an adult (>15 years) household member [adult leaves], the birth of a sibling [child arrives], and departure of a child (in almost all cases due to child death) [child departs].

The proportionality assumption that the hazard risk of out-migration is independent of time for any given variable was checked for key variables (e.g. access to taps, movement of other household members). The only significant interaction, that between time (length of exposure) and water point access, was included in the final models.

To control for any hierarchical structures in the data relating to the household, such as unmeasured environmental similarities between individuals within the same family, household random effects were entered. A total of 1912 young adults aged 15–30 years were included in the final model. Three models were estimated, the full sample (Model A, male only (Model B) and female only (Model C). MlwiN (Bristol) software version 2.24 was used to perform the statistical analyses.

## Results

Overall, 15.5% of young adults (15–30 years) had ever-migrated out from the villages; twice as many males as females (24% of males, 12.1% of females). Older individuals, those unmarried and childless and/or from wealthy families were all more likely to have migrated out (Table 2). The main stated reason for out-migration was seeking high school education (75%) or employment in casual wage-labour jobs (13%) in nearby towns of Derra and Iteya (see map in Figure 1). Other reasons given included accompanying family and visiting relatives. On average, migrants remained in urban centres for less than one year; those seeking education gaining only one extra school grade before returning to the village.

**Table 2:** Multilevel event history regression model for seasonal risk of out-migration (Odds ratio and CI), **p<0.05, *p<0.01.

	Model A	Model B	Model C
	All n = 1912	Male only n = 996	Female only n = 916
	15.5% migrants	24% migrants	12.1% migrants
	53436 exploded cases	27193 exploded cases	26243 exploded cases
**Fixed Effects**			
*Village*			
A	1.05 (0.67–1.64)	1.39 (0.77–2.49)	0.95 (0.46–2.08)
B	0.75 (0.44–1.28)	1.43 (0.73–2.83)	0.23 (0.08–0.68)**
C	0.84 (0.49–1.45)	1.29 (0.69–2.63)	0.57 (0.22–1.42)
D	0.71 (0.47–1.07)	0.77 (0.46–1.30)	0.65 (0.32–1.32)
E	Ref	Ref	Ref
**Household Level**			
*Religion*			
Orthodox Christian	Ref	Ref	Ref
Muslim	0.72 (0.7–1.03)	1.20 (0.76–1.90)	0.62 (0.33–1.17)
*Household wealth*			
Land holdings	1.23 (1.08–1.40)**	1.06 (0.89–1.26)	1.35 (1.07–1.70)**
Cattle herd size	1.02 (1.00–1.04)**	1.02 (0.99–1.04)**	1.01 (0.98–1.02)
*Household size*	1.02 (0.97–1.07)	0.96 (0.90–1.02)	1.07 (0.8–1.18)
*Sibship size*	1.00 (0.97–1.03)	1.05 (1.01–1.09)**	0.94 (0.88–1.01)*
*Random Effect- Household-level intercept*	1.16 (0.91–1.49)	1.14 (0.86–1.50)	1.21 (0.76–1.92)
*Education of head of household*			
Uneducated	Ref	Ref	Ref
Educated	1.04 (1.00–1.08)**	1.15 (1.09–1.20)**	0.95 (0.87–1.02)
**Individual Level**			
*Sex*			
Female	Ref		
Male	1.80 (1.38–2.36)**		
*Household status*			
Unmarried	Ref	Ref	
Married	0.61 (0.35–0.96)**	0.14 (0.07–0.25)**	
*Birth order*	1.02 (0.95–1.09)	0.99 (0.90–1.08)	1.07 (0.95–1.21)
**Time-varying Effects**			
*Time (Age)*	1.04 (1.01–1.05)**	1.05 (1.03–1.07)**	1.03 (1.01–1.05)**
*Access to development*	2.92 (1.63–4.90)**	2.97 (1.52–5.83)**	3.90 (1.69–9.01)**
*Child born/arrives*	1.99 (1.46–2.72)**	1.57 (1.08–2.28)**	2.36 (1.37–4.06)**
*Adult arrives*	0.26 (0.17–0.39)**	0.58 (0.33–0.99)**	0.16 (0.08–0.32)**
*Child dies/leaves*	0.90 (0.60–1.36)	0.93 (0.34–1.56)	1.08 (1.04–1.13)
*Adult leaves*	1.24 (0.94–1.65)	0.90 (0.64–1.28)	1.57 (0.96–2.55)
*Access to development×Time*	0.97 (0.82–0.98)**	0.97 (0.95–0.99)**	0.95 (0.92–0.98)**

**Figure 3:**
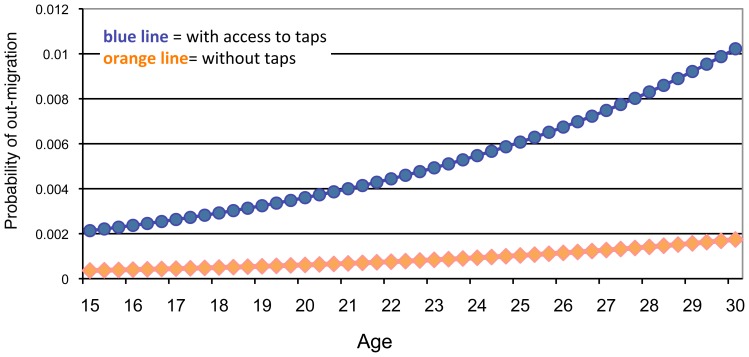
Predicted probability of out-migration of young adults as a function of access to water development (n = 1912, adults 15–30 years).

In the hazards regression analyses, the arrival of development intervention was associated with increased odds of out-migration for young adults (Table 2 and Figure 3). A young adult with access to taps was three times more likely to migrate to an urban centre compared to a young adult without access to taps in any given season (Table 2; access to taps odds ratio [OR] = 2.92, *p* = <0.05). These striking and immediate effects were found for both males and females. For all young adults, the positive effects of the development initiative on risk of out-migration increased with age (time×access to taps OR = 0.97, *p* = <0.05), indicating odds of out-migration did not diminish over time with exposure to the intervention.

Household dynamics, particularly the timing of the arrival of new births or new adult household members, were very important predictors of a young adult's relative risk of out-migration. The birth of a sibling doubled the odds of out-migration over time (OR = 1.99, *p* = <0.05). Conversely, the arrival of an adult to the household (in almost all cases a new spouse) immediately reduced these odds (OR = 0.26, *p* = <0.05). The departure of other household members (either through death in childhood, or out-migration), had no significant impact on out-migration of young adults. This suggests that in this population there is no indication of chain migration, or social influence of kin or other household members.

Wealthier individuals, those living in households with more land, and larger herd size, were all more likely to out-migrate. Interestingly, the sexes varied in their response to different forms of wealth. Males migrated from households with traditional forms of wealth (cattle herd size), and females were influenced by landholdings. Those individuals living in a household where the head of household had attended school, had increased odds of out-migration, particularly males. Household size did not appear to influence out-migration, and the effect of sibship size varied by sex. Large numbers of siblings increased the odds of out-migration for males, but reduced the odds of out-migration for females, indicative of extra sibling childcare and domestic responsibilities young unmarried females assume to support their kin in large families [30], [31]. Other household and village-level factors including religion and unobserved family-level effects did not strongly influence out-migration.

## Discussion

This investigation has identified a situation in which a rural development initiative has introduced unintended demographic consequences. We find an association between the recent installation of water taps in Southern Ethiopian villages, and the increased out-migration of young adults (15–30 years) to nearby urban centres. Young adults gaining access to taps were three times more likely to migrate out in a given season than their peers living without taps. This is most likely fuelled by increases in family sizes linked directly with the improved water supplies, which has immediately reduced child mortality and increased fertility [10]. Under conditions of population growth, out-migration of young adults may be one strategy to alleviate the harmful effects of increasing resource competition for scarce local resources (for food, agricultural land and marriage opportunities [17], [18]). This finding is supported by evidence that family dynamics predict the timing of out-migration. In our analyses, the birth of a younger sibling doubled the odds of a young adult migrating out to an urban centre. Males, who compete for heritable land, were particularly sensitive to family effects; large sibship size was a major determinant of male out-migration.

Secular trends that have arisen simultaneously with development intervention, for example improved roads, connectivity and aspirations of ‘modernity’ may contribute to a positive and generalised cultural outlook on migration. In the following discussion we focus on specific aspects of migration strategies in light of our findings.

### Migration as a strategy

Resource diversification is a key motivator for out-migration in this land-limited rural population. We found that most young migrants moved out in search of either high school education or off-farm employment opportunities in neighbouring urban centres. These towns, however, like many others in Ethiopia, currently struggle to provide the jobs, housing and services to support the growing demand [32]. Most of the young adults in our study returned to their villages within a year of migrating out.

Focus group discussions revealed that local opinion on the benefits of out-migration was divided. On one hand, there is optimism that migration will bring new livelihood opportunities; presenting an attractive alternative to diversify income in the face of land shortages. One villager reported “*If my children were not educated, they would live with me. But I don't have enough farming land, so I would have had a tough problem. Thanks to migration [for education] I am free from the problem*”. On the other, there is a growing understanding of financial hardships and other risks associated with urban living, including theft, exploitation and STDs. Stories of unsuccessful migration are commonly reported in the village. In the words of one village woman “*I know a girl who moved.…she told me that it is difficult to find work there [the city]. She felt ashamed to come back empty handed; without making any benefit to herself or her family. Without work, the life is difficult. She was facing great problems, but I told her to come back*”.

Only 13% of young migrants reported migrating out in search of employment. Male migrants sought casual construction work and females, jobs in domestic service, mainly in small nearby towns. However, jobs are commonly understood to be poorly paid and urban life, to be expensive. One villager reported *“We see people moving [to towns], but we haven't seen them sending something [remittances] back.…if they are successful they should send something back. They are facing even more problems than they had faced here [in the villages]”*. Our data reveal that urban migrants were more likely to make financial contributions to the rural household than their non-migrant peers who remained to work on farms (27.5% versus 15.5%). However remittances are no larger than agricultural earnings: the average migrant sent 50 USD over a 3 month season (Table S1). Maintaining links with their villages by sending these small gifts of money may be a form of self-insurance, representing one response to rising risk exposure in urban centres [33].

The majority of rural to urban Arsi Oromo migrants reported that they were migrating in search of high school qualifications, which are unavailable locally. Despite urban education incurring significant costs in terms of money (for accommodation, food and stationery) and time spent away from productive agricultural activities, the demand for advanced school qualifications has recently increased [13]. Educated individuals, particularly males, achieve elevated social status and greater success in securing high status marriages when they return to the village, reflected in large marriage payments. At marriage, high school educated males command twice the brideprice (from their kin) and receive triple the dowry (from their in-laws) compared with their less educated peers (Gibson, in prep). While few-off farm or skilled job opportunities exist in rural areas, urban education is becoming an important signal of status in a society where inequalities in material wealth (land and cattle) are relatively small due to the levelling effects of 30 years of land redistribution and periodic crop failure [34]. More broadly, education is understood as being important for human development, as it is associated with better health, economic growth and prosperity [35].

Young female rural-urban migration is currently relatively uncommon (only 12.1% females migrated out), due to their domestic responsibilities, most notably caring for younger siblings. We predict, however, that levels of female out-migration will increase in the future, due to the growing opportunities for income generation as domestic servants, particularly in the Middle East and Sudan [36]. Young migrant females in our sample made equal financial contributions to rural households compared with migrant males, but considerably more than their female peers who remained in unpaid domestic work in the villages (of whom <3% contribute financially to the household). Short-term urban employment may become an attractive alternative to unpaid rural domestic work, offering some young women the chance to gain an income, status and autonomy when they return to the village (e.g. choice of marriage partners and bargaining power within marriage). Furthermore, a growing trend towards smaller family sizes may reduce the child-care labour demands on women's time.

In summary, at the household and individual level, migration currently represents a short-term strategy for alleviating resource competition between siblings, particularly males. Further, we identify that a water intervention scheme which has underpinned increases in family size may be fuelling this movement. While levels of out-migration and financial returns are currently low and residence temporary, a continued decline in the ratio of agricultural land to people and environmental degradation in rural areas suggest increases of all three in the future.

### The demography of development

This study has relevance for development policy-makers and users. If population growth associated with development is fuelling recent increases in rural to urban migration, then additional pressure may be exerted on already stretched rural and urban services [5], [32]. Ethiopian Government statistics indicate that up to 50% of the urban population are landless migrants from rural villages [37]. However, jobs, housing, and services are not available to sustain this migrant community (a third of urban dwellers are unemployed and 80% live in substandard housing [4]).

Over the longer term, increased urban growth and urbanisation may lead to more positive outcomes. For example, urbanisation may speed up fertility decline, as the rising cost of raising successful children in urban skill-based economies should drive a preference for small family sizes [38], [39]. Gurmu and Mace [40] have shown that female migrants to Addis Ababa delay reproduction and limit fertility in response to higher cost of urban living, while a study in Mozambique found that rural women married to successful migrants are more likely to use contraceptives [41]. In the study villages there is already a growing demand for “modern” family planning, with uptake increasing from <1% to 19% over the last 10 years [42]. However, supplies are limited. Arsi Oromo women, like many across the rural developing world, remain without adequate family planning provision. It seems clear that the priorities for development policy-makers should involve planning to manage increased urban growth, complemented by efforts to reduce unwanted pregnancies in rural areas.

The demographic consequences of rural intervention initiatives are rarely considered, but it is imperative that they should be, not least as a means of assessing their long-term and wider effectiveness. Population growth, environmental degradation and unplanned urban growth have been highlighted as the main factors impeding sustainable global social and economic development [43]. However, disentangling the independent influence of these processes is not easy [44]. If the key challenges of the 21^st^ century relate to population pressures, we need to develop a better understanding of the relationship between demography and development.

## Supporting Information

Table S1 **Sample descriptive statistics.**
(DOC)
