# Supplementary material for: Rural to Urban Migration Is an Unforeseen Impact of Development Intervention in Ethiopia
Source: PLoS One. 2012 Nov 14;7(11):e48708. doi: 10.1371/journal.pone.0048708 (PMC3498254; doi:10.1371/journal.pone.0048708)
Supplement: Table S1 — Sample descriptive statistics. (DOC) [file pone.0048708.s001.doc]

|  | % of all adults 15-30 | Mean value for all adults 15-30 | % of migrants | Mean value for migrants |
| --- | --- | --- | --- | --- |
| *Village* |  |  |  |  |
| A | 22.9 |  | 20.1 |  |
| B | 17.2 |  | 11.7 |  |
| C | 19.8 |  | 22.5 |  |
| D | 22.3 |  | 19.5 |  |
| E | 17.9 |  | 26.3 |  |
| *Religion* |  |  |  |  |
| Orthodox Christian | 17.1 |  | 20.4 |  |
| Muslim | 82.9 |  | 79.6 |  |
| *Household wealth* |  |  |  |  |
| Landless | 87 |  | 89.5 |  |
| Landholding size of head of household |  | 1.39 (±0.95) |  | 1.75 (±0.98) |
| *Household size* |  | 6.54 (±3.02) |  | 7.50 (±3.14) |
| *Sibship size* |  | 10.17 (±4.26) |  | 10.81(±4.07) |
| *Education* |  |  |  |  |
| Uneducated | 19.4 |  | 3.6 |  |
| Head of household is uneducated | 39.9 |  | 46.8 |  |
| *Sex* |  |  |  |  |
| Male | 52.1 |  | 66.8 |  |
| *Marital status* |  |  |  |  |
| Unmarried | 59.3 |  | 83.5 |  |
| Childless | 65 |  | 88.3 |  |
| *Birth order* |  | 4.13 (±2.01) |  | 4.14 (±2.04) |
| *Age* |  | 22.41 (±4.48) |  | 23.06 (±3.74) |
| *Income in last 3 months* |  |  |  |  |
| % contributing any earnings | 15.5 |  | 27.5 |  |
| Mean earnings (USD) |  | 53 (49) |  | 49 (50) |
